# Stem Cells Applications in Regenerative Medicine and Disease Therapeutics

**DOI:** 10.1155/2016/6940283

**Published:** 2016-07-19

**Authors:** Ranjeet Singh Mahla

**Affiliations:** Department of Biological Sciences, Indian Institute of Science Education and Research (IISER), Bhopal, Madhya Pradesh 462066, India

## Abstract

Regenerative medicine, the most recent and emerging branch of medical science, deals with functional restoration of tissues or organs for the patient suffering from severe injuries or chronic disease. The spectacular progress in the field of stem cell research has laid the foundation for cell based therapies of disease which cannot be cured by conventional medicines. The indefinite self-renewal and potential to differentiate into other types of cells represent stem cells as frontiers of regenerative medicine. The transdifferentiating potential of stem cells varies with source and according to that regenerative applications also change. Advancements in gene editing and tissue engineering technology have endorsed the ex vivo remodelling of stem cells grown into 3D organoids and tissue structures for personalized applications. This review outlines the most recent advancement in transplantation and tissue engineering technologies of ESCs, TSPSCs, MSCs, UCSCs, BMSCs, and iPSCs in regenerative medicine. Additionally, this review also discusses stem cells regenerative application in wildlife conservation.

## 1. Introduction

**Figure 1:**
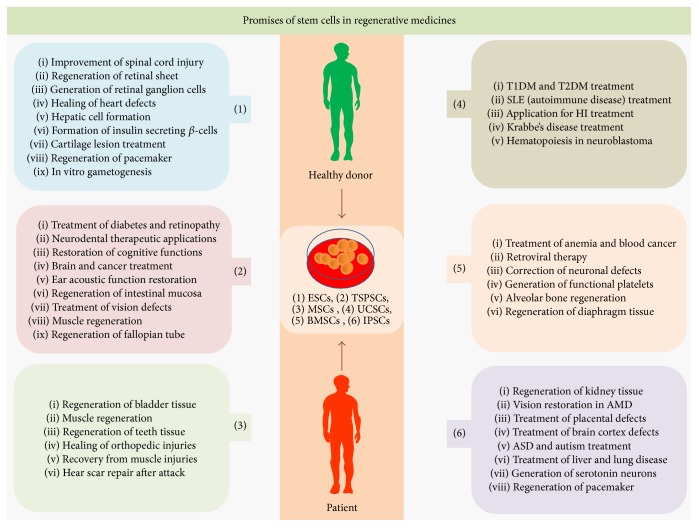
Promises of stem cells in regenerative medicine: the six classes of stem cells, that is, embryonic stem cells (ESCs), tissue specific progenitor stem cells (TSPSCs), mesenchymal stem cells (MSCs), umbilical cord stem cells (UCSCs), bone marrow stem cells (BMSCs), and induced pluripotent stem cells (iPSCs), have many promises in regenerative medicine and disease therapeutics.

**Figure 2 fig2:**
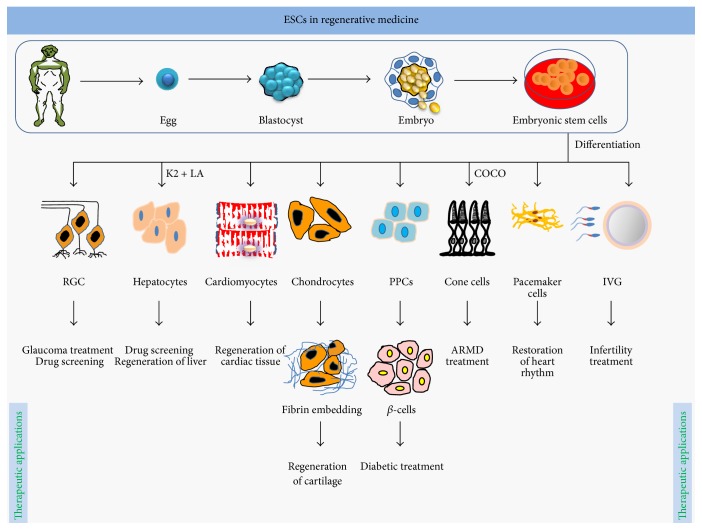
ESCs in regenerative medicine: ESCs, sourced from ICM of gastrula, have tremendous promises in regenerative medicine. These cells can differentiate into more than 200 types of cells representing three germ layers. With defined culture conditions, ESCs can be transformed into hepatocytes, retinal ganglion cells, chondrocytes, pancreatic progenitor cells, cone cells, cardiomyocytes, pacemaker cells, eggs, and sperms which can be used in regeneration of tissue and treatment of disease in tissue specific manner.

**Figure 3 fig3:**
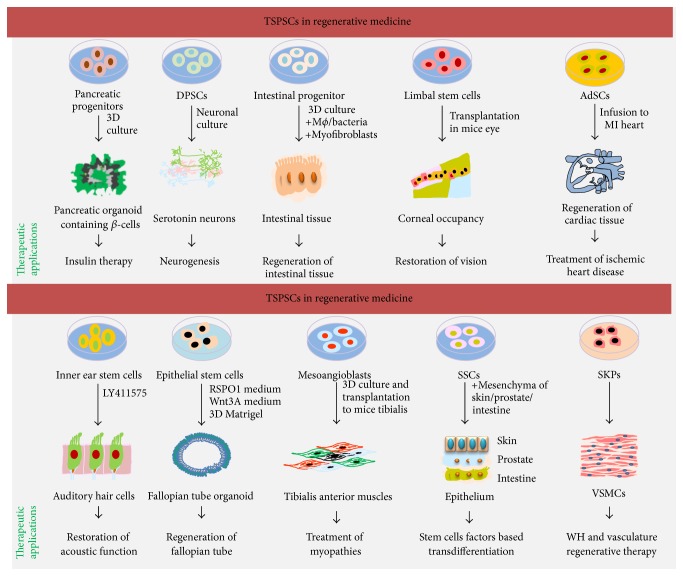
TSPSCs in regenerative medicine: tissue specific stem and progenitor cells have potential to differentiate into other cells of the tissue. Characteristically inner ear stem cells can be transformed into auditory hair cells, skin progenitors into vascular smooth muscle cells, mesoangioblasts into tibialis anterior muscles, and dental pulp stem cells into serotonin cells. The 3D-culture of TSPSCs in complex biomaterial gives rise to tissue organoids, such as pancreatic organoid from pancreatic progenitor, intestinal tissue organoids from intestinal progenitor cells, and fallopian tube organoids from fallopian tube epithelial cells. Transplantation of TSPSCs regenerates targets tissue such as regeneration of tibialis muscles from mesoangioblasts, cardiac tissue from AdSCs, and corneal tissue from limbal stem cells. Cell growth and transformation factors secreted by TSPSCs can change cells fate to become other types of cell, such that SSCs coculture with skin, prostate, and intestine mesenchyme transforms these cells from MSCs into epithelial cells fate.

**Figure 4 fig4:**
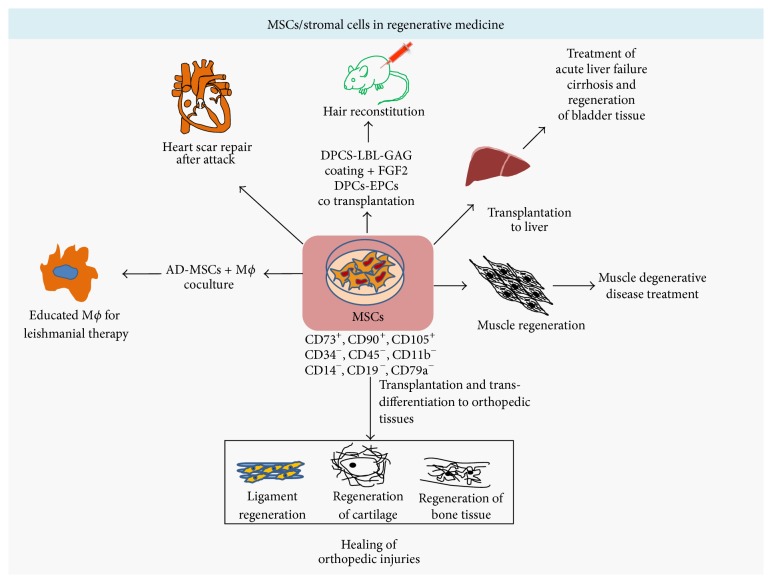
MSCs in regenerative medicine: mesenchymal stem cells are CD73^+^, CD90^+^, CD105^+^, CD34^−^, CD45^−^, CD11b^−^, CD14^−^, CD19^−^, and CD79a^−^ cells, also known as stromal cells. These bodily MSCs represented here do not account for MSCs of bone marrow and umbilical cord. Upon transplantation and transdifferentiation these bodily MSCs regenerate into cartilage, bones, and muscles tissue. Heart scar formed after heart attack and liver cirrhosis can be treated from MSCs. ECM coating provides the niche environment for MSCs to regenerate into hair follicle, stimulating hair growth.

**Figure 5 fig5:**
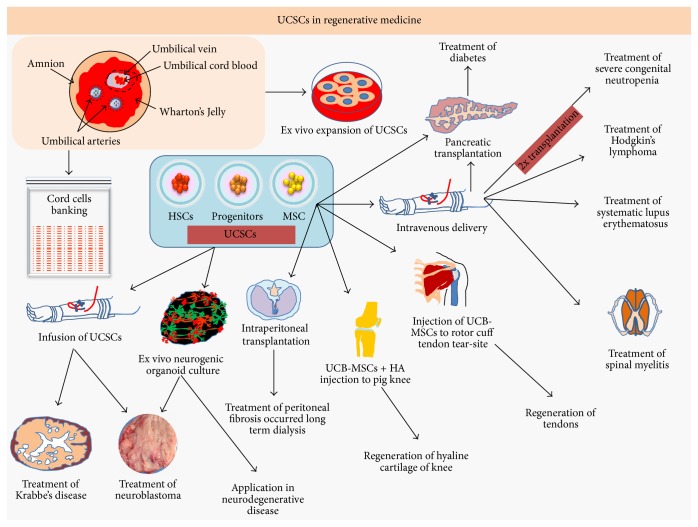
UCSCs in regenerative medicine: umbilical cord, the readily available source of stem cells, has emerged as futuristic source for personalized stem cell therapy. Transplantation of UCSCs to Krabbe's disease patients regenerates myelin tissue and recovers neuroblastoma patients through restoring tissue homeostasis. The UCSCs organoids are readily available tissue source for treatment of neurodegenerative disease. Peritoneal fibrosis caused by long term dialysis, tendon tissue degeneration, and defective hyaline cartilage can be regenerated by UCSCs. Intravenous injection of UCSCs enables treatment of diabetes, spinal myelitis, systemic lupus erythematosus, Hodgkin's lymphoma, and congenital neuropathies. Cord blood stem cells banking avails long lasting source of stem cells for personalized therapy and regenerative medicine.

**Figure 6 fig6:**
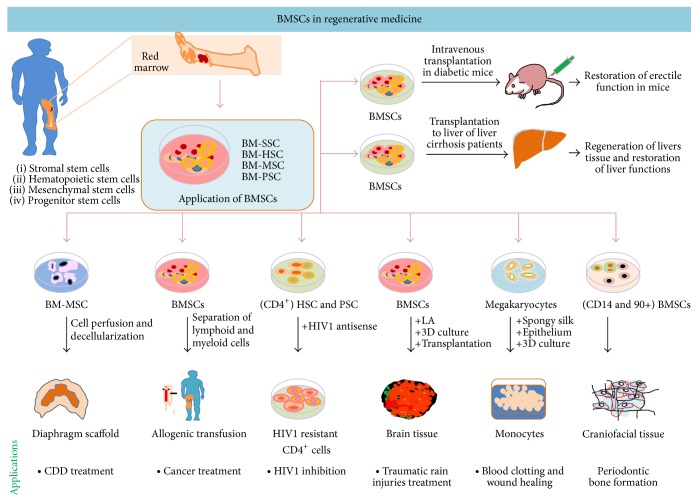
BMSCs in regenerative medicine: bone marrow, the soft sponge bone tissue that consisted of stromal, hematopoietic, and mesenchymal and progenitor stem cells, is responsible for blood formation. Even halo-HLA matched BMSCs can cure from disease and regenerate tissue. BMSCs can regenerate craniofacial tissue, brain tissue, diaphragm tissue, and liver tissue and restore erectile function and transdifferentiation monocytes. These multipotent stem cells can cure host from cancer and infection of HIV and HCV.

**Figure 7 fig7:**
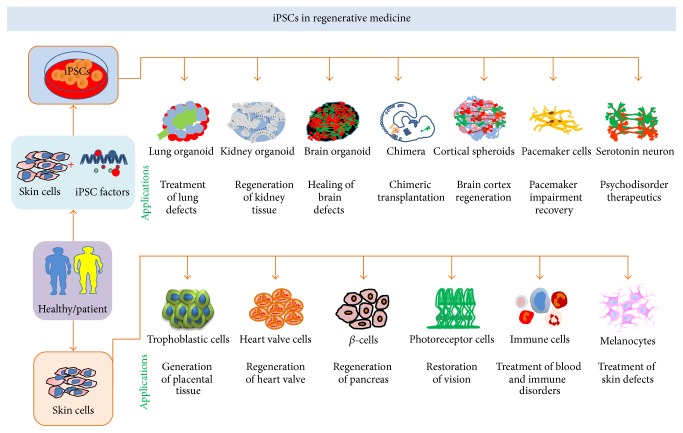
iPSCs in regenerative medicine: using the edge of iPSCs technology, skin fibroblasts and other adult tissues derived, terminally differentiated cells can be transformed into ESCs-like cells. It is possible that adult cells can be transformed into cells of distinct lineages bypassing the phase of pluripotency. The tissue specific defined culture can transform skin cells to become trophoblast, heart valve cells, photoreceptor cells, immune cells, melanocytes, and so forth. ECM complexation with iPSCs enables generation of tissue organoids for lung, kidney, brain, and other organs of the body. Similar to ESCs, iPSCs also can be transformed into cells representing three germ layers such as pacemaker cells and serotonin cells.

**Figure 8:**
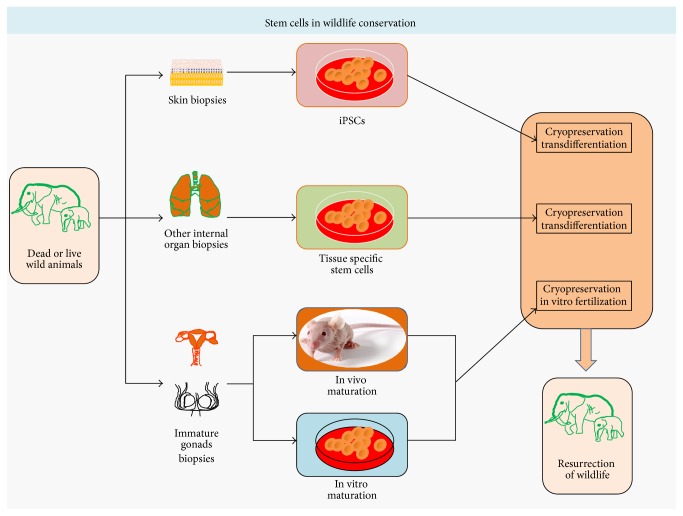
Stem cells in wildlife conservation: tissue biopsies obtained from dead and live wild animals can be either cryopreserved or transdifferentiated to other types of cells, through culture in defined culture medium or in vivo maturation. Stem cells and adult tissue derived iPSCs have great potential of regenerative medicine and disease therapeutics. Gonadal tissue procured from dead wild animals can be matured, ex vivo and in vivo for generation of sperm and egg, which can be used for assistive reproductive technology oriented captive breeding of wild animals or even for resurrection of wildlife.

Regenerative medicine, the most recent and emerging branch of medical science, deals with functional restoration of specific tissue and/or organ of the patients suffering with severe injuries or chronic disease conditions, in the state where bodies own regenerative responses do not suffice [1]. In the present scenario donated tissues and organs cannot meet the transplantation demands of aged and diseased populations that have driven the thrust for search for the alternatives. Stem cells are endorsed with indefinite cell division potential, can transdifferentiate into other types of cells, and have emerged as frontline regenerative medicine source in recent time, for reparation of tissues and organs anomalies occurring due to congenital defects, disease, and age associated effects [1]. Stem cells pave foundation for all tissue and organ system of the body and mediates diverse role in disease progression, development, and tissue repair processes in host. On the basis of transdifferentiation potential, stem cells are of four types, that is, (1) unipotent, (2) multipotent, (3) pluripotent, and (4) totipotent [2]. Zygote, the only totipotent stem cell in human body, can give rise to whole organism through the process of transdifferentiation, while cells from inner cells mass (ICM) of embryo are pluripotent in their nature and can differentiate into cells representing three germ layers but do not differentiate into cells of extraembryonic tissue [2]. Stemness and transdifferentiation potential of the embryonic, extraembryonic, fetal, or adult stem cells depend on functional status of pluripotency factors like OCT4, cMYC, KLF44, NANOG, SOX2, and so forth [3–5]. Ectopic expression or functional restoration of endogenous pluripotency factors epigenetically transforms terminally differentiated cells into ESCs-like cells [3], known as induced pluripotent stem cells (iPSCs) [3, 4]. On the basis of regenerative applications, stem cells can be categorized as embryonic stem cells (ESCs), tissue specific progenitor stem cells (TSPSCs), mesenchymal stem cells (MSCs), umbilical cord stem cells (UCSCs), bone marrow stem cells (BMSCs), and iPSCs (Figure 1; Table 1). The transplantation of stem cells can be autologous, allogenic, and syngeneic for induction of tissue regeneration and immunolysis of pathogen or malignant cells. For avoiding the consequences of host-versus-graft rejections, tissue typing of human leucocyte antigens (HLA) for tissue and organ transplant as well as use of immune suppressant is recommended [6]. Stem cells express major histocompatibility complex (MHC) receptor in low and secret chemokine that recruitment of endothelial and immune cells is enabling tissue tolerance at graft site [6]. The current stem cell regenerative medicine approaches are founded onto tissue engineering technologies that combine the principles of cell transplantation, material science, and microengineering for development of organoid; those can be used for physiological restoration of damaged tissue and organs. The tissue engineering technology generates nascent tissue on biodegradable 3D-scaffolds [7, 8]. The ideal scaffolds support cell adhesion and ingrowths, mimic mechanics of target tissue, support angiogenesis and neovascularisation for appropriate tissue perfusion, and, being nonimmunogenic to host, do not require systemic immune suppressant [9]. Stem cells number in tissue transplant impacts upon regenerative outcome [10]; in that case prior ex vivo expansion of transplantable stem cells is required [11]. For successful regenerative outcomes, transplanted stem cells must survive, proliferate, and differentiate in site specific manner and integrate into host circulatory system [12]. This review provides framework of most recent (Table 1; Figures 1 [Fig fig2] [Fig fig3] [Fig fig4] [Fig fig5] [Fig fig6] [Fig fig7]–8) advancement in transplantation and tissue engineering technologies of ESCs, TSPSCs, MSCs, UCSCs, BMSCs, and iPSCs in regenerative medicine. Additionally, this review also discusses stem cells as the tool of regenerative applications in wildlife conservation.

## 2. ESCs in Regenerative Medicine

For the first time in 1998, Thomson isolated human ESCs (hESCs) [13]. ESCs are pluripotent in their nature and can give rise to more than 200 types of cells and promises for the treatment of any kinds of disease [13]. The pluripotency fate of ESCs is governed by functional dynamics of transcription factors OCT4, SOX2, NANOG, and so forth, which are termed as pluripotency factors. The two alleles of the OCT4 are held apart in pluripotency state in ESCs; phase through homologues pairing during embryogenesis and transdifferentiation processes [14] has been considered as critical regulatory switch for lineage commitment of ESCs. The diverse lineage commitment potential represents ESCs as ideal model for regenerative therapeutics of disease and tissue anomalies. This section of review on ESCs discusses transplantation and transdifferentiation of ESCs into retinal ganglion, hepatocytes, cardiomyocytes, pancreatic progenitors, chondrocytes, cones, egg sperm, and pacemaker cells (Figure 2; Table 1). Infection, cancer treatment, and accidents can cause spinal cord injuries (SCIs). The transplantation of hESCs to paraplegic or quadriplegic SCI patients improves body control, balance, sensation, and limbal movements [15], where transplanted stem cells do homing to injury sites. By birth, humans have fixed numbers of cone cells; degeneration of retinal pigment epithelium (RPE) of macula in central retina causes age-related macular degeneration (ARMD). The genomic incorporation of COCO gene (expressed during embryogenesis) in the developing embryo leads lineage commitment of ESCs into cone cells, through suppression of TGF*β*, BMP, and Wnt signalling pathways. Transplantation of these cone cells to eye recovers individual from ARMD phenomenon, where transplanted cone cells migrate and form sheet-like structure in host retina [16]. However, establishment of missing neuronal connection of retinal ganglion cells (RGCs), cones, and PRE is the most challenging aspect of ARMD therapeutics. Recently, Donald Z Jacks group at John Hopkins University School of Medicine has generated RGCs from CRISPER-Cas9-m-Cherry reporter ESCs [17]. During ESCs transdifferentiation process, CRIPER-Cas9 directs the knock-in of m-Cherry reporter into 3′UTR of BRN3B gene, which is specifically expressed in RGCs and can be used for purification of generated RGCs from other cells [17]. Furthermore, incorporation of forskolin in transdifferentiation regime boosts generation of RGCs. Coaxing of these RGCs into biomaterial scaffolds directs axonal differentiation of RGCs. Further modification in RGCs generation regime and composition of biomaterial scaffolds might enable restoration of vision for ARMD and glaucoma patients [17]. Globally, especially in India, cardiovascular problems are a more common cause of human death, where biomedical therapeutics require immediate restoration of heart functions for the very survival of the patient. Regeneration of cardiac tissue can be achieved by transplantation of cardiomyocytes, ESCs-derived cardiovascular progenitors, and bone marrow derived mononuclear cells (BMDMNCs); however healing by cardiomyocytes and progenitor cells is superior to BMDMNCs but mature cardiomyocytes have higher tissue healing potential, suppress heart arrhythmias, couple electromagnetically into hearts functions, and provide mechanical and electrical repair without any associated tumorigenic effects [18, 19]. Like CM differentiation, ESCs derived liver stem cells can be transformed into Cytp450-hepatocytes, mediating chemical modification and catabolism of toxic xenobiotic drugs [20]. Even today, availability and variability of functional hepatocytes are a major a challenge for testing drug toxicity [20]. Stimulation of ESCs and ex vivo VitK12 and lithocholic acid (a by-product of intestinal flora regulating drug metabolism during infancy) activates pregnane X receptor (PXR), CYP3A4, and CYP2C9, which leads to differentiation of ESCs into hepatocytes; those are functionally similar to primary hepatocytes, for their ability to produce albumin and apolipoprotein B100 [20]. These hepatocytes are excellent source for the endpoint screening of drugs for accurate prediction of clinical outcomes [20]. Generation of hepatic cells from ESCs can be achieved in multiple ways, as serum-free differentiation [21], chemical approaches [20, 22], and genetic transformation [23, 24]. These ESCs-derived hepatocytes are long lasting source for treatment of liver injuries and high throughput screening of drugs [20, 23, 24]. Transplantation of the inert biomaterial encapsulated hESCs-derived pancreatic progenitors (CD24^+^, CD49^+^, and CD133^+^) differentiates into *β*-cells, minimizing high fat diet induced glycemic and obesity effects in mice [25] (Table 1). Addition of antidiabetic drugs into transdifferentiation regime can boost ESCs conservation into *β*-cells [25], which theoretically can cure T2DM permanently [25]. ESCs can be differentiated directly into insulin secreting *β*-cells (marked with GLUT2, INS1, GCK, and PDX1) which can be achieved through PDX1 mediated epigenetic reprogramming [26]. Globally, osteoarthritis affects millions of people and occurs when cartilage at joints wears away, causing stiffness of the joints. The available therapeutics for arthritis relieve symptoms but do not initiate reverse generation of cartilage. For young individuals and athletes replacement of joints is not feasible like old populations; in that case transplantation of stem cells represents an alternative for healing cartilage injuries [27]. Chondrocytes, the cartilage forming cells derived from hESC, embedded in fibrin gel effectively heal defective cartilage within 12 weeks, when transplanted to focal cartilage defects of knee joints in mice without any negative effect [27]. Transplanted chondrocytes form cell aggregates, positive for SOX9 and collagen II, and defined chondrocytes are active for more than 12 wks at transplantation site, advocating clinical suitability of chondrocytes for treatment of cartilage lesions [27]. The integrity of ESCs to integrate and differentiate into electrophysiologically active cells provides a means for natural regulation of heart rhythm as biological pacemaker. Coaxing of ESCs into inert biomaterial as well as propagation in defined culture conditions leads to transdifferentiation of ESCs to become sinoatrial node (SAN) pacemaker cells (PCs) [28]. Genomic incorporation TBox3 into ESCs ex vivo leads to generation of PCs-like cells; those express activated leukocyte cells adhesion molecules (ALCAM) and exhibit similarity to PCs for gene expression and immune functions [28]. Transplantation of PCs can restore pacemaker functions of the ailing heart [28]. In summary, ESCs can be transdifferentiated into any kinds of cells representing three germ layers of the body, being most promising source of regenerative medicine for tissue regeneration and disease therapy (Table 1). Ethical concerns limit the applications of ESCs, where set guidelines need to be followed; in that case TSPSCs, MSCs, UCSCs, BMSCs, and iPSCs can be explored as alternatives.

## 3. TSPSCs in Regenerative Medicine

TSPSCs maintain tissue homeostasis through continuous cell division, but, unlike ESCs, TSPSCs retain stem cells plasticity and differentiation in tissue specific manner, giving rise to few types of cells (Table 1). The number of TSPSCs population to total cells population is too low; in that case their harvesting as well as in vitro manipulation is really a tricky task [29], to explore them for therapeutic scale. Human body has foundation from various types of TSPSCs; discussing the therapeutic application for all types is not feasible. This section of review discusses therapeutic application of pancreatic progenitor cells (PPCs), dental pulp stem cells (DPSCs), inner ear stem cells (IESCs), intestinal progenitor cells (IPCs), limbal progenitor stem cells (LPSCs), epithelial progenitor stem cells (EPSCs), mesoangioblasts (MABs), spermatogonial stem cells (SSCs), the skin derived precursors (SKPs), and adipose derived stem cells (AdSCs) (Figure 3; Table 1). During embryogenesis PPCs give rise to insulin-producing *β*-cells. The differentiation of PPCs to become *β*-cells is negatively regulated by insulin [30]. PPCs require active FGF and Notch signalling; growing more rapidly in community than in single cell populations advocates the functional importance of niche effect in self-renewal and transdifferentiation processes. In 3D-scaffold culture system, mice embryo derived PPCs grow into hollow organoid spheres; those finally differentiate into insulin-producing *β*-cell clusters [29]. The DSPSCs, responsible for maintenance of teeth health status, can be sourced from apical papilla, deciduous teeth, dental follicle, and periodontal ligaments, have emerged as regenerative medicine candidate, and might be explored for treatment of various kinds of disease including restoration neurogenic functions in teeth [31, 32]. Expansion of DSPSCs in chemically defined neuronal culture medium transforms them into a mixed population of cholinergic, GABAergic, and glutaminergic neurons; those are known to respond towards acetylcholine, GABA, and glutamine stimulations in vivo. These transformed neuronal cells express nestin, glial fibrillary acidic protein (GFAP), *β*III-tubulin, and voltage gated L-type Ca^2+^ channels [32]. However, absence of Na^+^ and K^+^ channels does not support spontaneous action potential generation, necessary for response generation against environmental stimulus. All together, these primordial neuronal stem cells have possible therapeutic potential for treatment of neurodental problems [32]. Sometimes, brain tumor chemotherapy can cause neurodegeneration mediated cognitive impairment, a condition known as chemobrain [33]. The intrahippocampal transplantation of human derived neuronal stem cells to cyclophosphamide behavioural decremented mice restores cognitive functions in a month time. Here the transplanted stem cells differentiate into neuronal and astroglial lineage, reduce neuroinflammation, and restore microglial functions [33]. Furthermore, transplantation of stem cells, followed by chemotherapy, directs pyramidal and granule-cell neurons of the gyrus and CA1 subfields of hippocampus which leads to reduction in spine and dendritic cell density in the brain. These findings suggest that transplantation of stem cells to cranium restores cognitive functions of the chemobrain [33]. The hair cells of the auditory system produced during development are not postmitotic; loss of hair cells cannot be replaced by inner ear stem cells, due to active state of the Notch signalling [34]. Stimulation of inner ear progenitors with *ϒ*-secretase inhibitor (LY411575) abrogates Notch signalling through activation of transcription factor atonal homologue 1 (Atoh1) and directs transdifferentiation of progenitors into cochlear hair cells [34]. Transplantation of in vitro generated hair cells restores acoustic functions in mice, which can be the potential regenerative medicine candidates for the treatment of deafness [34]. Generation of the hair cells also can be achieved through overexpression of *β*-catenin and Atoh1 in Lrg5^+^ cells in vivo [35]. Similar to ear progenitors, intestine of the digestive tract also has its own tissue specific progenitor stem cells, mediating regeneration of the intestinal tissue [34, 36]. Dysregulation of the common stem cells signalling pathways, Notch/BMP/TGF-*β*/Wnt, in the intestinal tissue leads to disease. Information on these signalling pathways [37] is critically important in designing therapeutics. Coaxing of the intestinal tissue specific progenitors with immune cells (macrophages), connective tissue cells (myofibroblasts), and probiotic bacteria into 3D-scaffolds of inert biomaterial, crafting biological environment, is suitable for differentiation of progenitors to occupy the crypt-villi structures into these scaffolds [36]. Omental implementation of these crypt-villi structures to dogs enhances intestinal mucosa through regeneration of goblet cells containing intestinal tissue [36]. These intestinal scaffolds are close approach for generation of implantable intestinal tissue, divested by infection, trauma, cancer, necrotizing enterocolitis (NEC), and so forth [36]. In vitro culture conditions cause differentiation of intestinal stem cells to become other types of cells, whereas incorporation of valproic acid and CHIR-99021 in culture conditions avoids differentiation of intestinal stem cells, enabling generation of indefinite pool of stem cells to be used for regenerative applications [38]. The limbal stem cells of the basal limbal epithelium, marked with ABCB5, are essential for regeneration and maintenance of corneal tissue [39]. Functional status of ABCB5 is critical for survival and functional integrity of limbal stem cells, protecting them from apoptotic cell death [39]. Limbal stem cells deficiency leads to replacement of corneal epithelium with visually dead conjunctival tissue, which can be contributed by burns, inflammation, and genetic factors [40]. Transplanted human cornea stem cells to mice regrown into fully functional human cornea, possibly supported by blood eye barrier phenomena, can be used for treatment of eye diseases, where regeneration of corneal tissue is critically required for vision restoration [39]. Muscle degenerative disease like duchenne muscular dystrophy (DMD) can cause extensive thrashing of muscle tissue, where tissue engineering technology can be deployed for functional restoration of tissue through regeneration [41]. Encapsulation of mouse or human derived MABs (engineered to express placental derived growth factor (PDGF)) into polyethylene glycol (PEG) fibrinogen hydrogel and their transplantation beneath the skin at ablated tibialis anterior form artificial muscles, which are functionally similar to those of normal tibialis anterior muscles [41]. The PDGF attracts various cell types of vasculogenic and neurogenic potential to the site of transplantation, supporting transdifferentiation of mesoangioblasts to become muscle fibrils [41]. The therapeutic application of MABs in skeletal muscle regeneration and other therapeutic outcomes has been reviewed by others [42]. One of the most important tissue specific stem cells, the male germline stem cells or spermatogonial stem cells (SSCs), produces spermatogenic lineage through mesenchymal and epithets cells [43] which itself creates niche effect on other cells. In vivo transplantation of SSCs with prostate, skin, and uterine mesenchyme leads to differentiation of these cells to become epithelia of the tissue of origin [43]. These newly formed tissues exhibit all physical and physiological characteristics of prostate and skin and the physical characteristics of prostate, skin, and uterus, express tissue specific markers, and suggest that factors secreted from SSCs lead to lineage conservation which defines the importance of niche effect in regenerative medicine [43]. According to an estimate, more than 100 million people are suffering from the condition of diabetic retinopathy, a progressive dropout of vascularisation in retina that leads to loss of vision [44]. The intravitreal injection of adipose derived stem cells (AdSCs) to the eye restores microvascular capillary bed in mice. The AdSCs from healthy donor produce higher amounts of vasoprotective factors compared to glycemic mice, enabling superior vascularisation [44]. However use of AdSCs for disease therapeutics needs further standardization for cell counts in dose of transplant and monitoring of therapeutic outcomes at population scale [44]. Apart from AdSCs, other kinds of stem cells also have therapeutic potential in regenerative medicine for treatment of eye defects, which has been reviewed by others [45]. Fallopian tubes, connecting ovaries to uterus, are the sites where fertilization of the egg takes place. Infection in fallopian tubes can lead to inflammation, tissue scarring, and closure of the fallopian tube which often leads to infertility and ectopic pregnancies. Fallopian is also the site where onset of ovarian cancer takes place. The studies on origin and etiology of ovarian cancer are restricted due to lack of technical advancement for culture of epithelial cells. The in vitro 3D organoid culture of clinically obtained fallopian tube epithelial cells retains their tissue specificity, keeps cells alive, which differentiate into typical ciliated and secretory cells of fallopian tube, and advocates that ectopic examination of fallopian tube in organoid culture settings might be the ideal approach for screening of cancer [46]. The sustained growth and differentiation of fallopian TSPSCs into fallopian tube organoid depend both on the active state of the Wnt and on paracrine Notch signalling [46]. Similar to fallopian tube stem cells, subcutaneous visceral tissue specific cardiac adipose (CA) derived stem cells (AdSCs) have the potential of differentiation into cardiovascular tissue [47]. Systemic infusion of CA-AdSCs into ischemic myocardium of mice regenerates heart tissue and improves cardiac function through differentiation to endothelial cells, vascular smooth cells, and cardiomyocytes and vascular smooth cells. The differentiation and heart regeneration potential of CA-AdSCs are higher than AdSCs [48], representing CA-AdSCs as potent regenerative medicine candidates for myocardial ischemic therapy [47]. The skin derived precursors (SKPs), the progenitors of dermal papilla/hair/hair sheath, give rise to multiple tissues of mesodermal and/or ectodermal origin such as neurons, Schwann cells, adipocytes, chondrocytes, and vascular smooth muscle cells (VSMCs). VSMCs mediate wound healing and angiogenesis process can be derived from human foreskin progenitor SKPs, suggesting that SKPs derived VSMCs are potential regenerative medicine candidates for wound healing and vasculature injuries treatments [49]. In summary, TSPSCs are potentiated with tissue regeneration, where advancement in organoid culture (Figure 3; Table 1) technologies defines the importance of niche effect in tissue regeneration and therapeutic outcomes of ex vivo expanded stem cells.

## 4. MSCs/Stromal Cells in Regenerative Medicine

MSCs, the multilineage stem cells, differentiate only to tissue of mesodermal origin, which includes tendons, bone, cartilage, ligaments, muscles, and neurons [50]. MSCs are the cells which express combination of markers: CD73^+^, CD90^+^, CD105^+^, CD11b^−^, CD14^−^, CD19^−^, CD34^−^, CD45^−^, CD79a^−^, and HLA-DR, reviewed elsewhere [50]. The application of MSCs in regenerative medicine can be generalized from ongoing clinical trials, phasing through different state of completions, reviewed elsewhere [90]. This section of review outlines the most recent representative applications of MSCs (Figure 4; Table 1). The anatomical and physiological characteristics of both donor and receiver have equal impact on therapeutic outcomes. The bone marrow derived MSCs (BMDMSCs) from baboon are morphologically and phenotypically similar to those of bladder stem cells and can be used in regeneration of bladder tissue. The BMDMSCs (CD105^+^, CD73^+^, CD34^−^, and CD45^−^), expressing GFP reporter, coaxed with small intestinal submucosa (SIS) scaffolds, augment healing of degenerated bladder tissue within 10 wks of the transplantation [51]. The combinatorial CD characterized MACs are functionally active at transplantation site, which suggests that CD characterization of donor MSCs yields superior regenerative outcomes [51]. MSCs also have potential to regenerate liver tissue and treat liver cirrhosis, reviewed elsewhere [91]. The regenerative medicinal application of MSCs utilizes cells in two formats as direct transplantation or first transdifferentiation and then transplantation; ex vivo transdifferentiation of MSCs deploys retroviral delivery system that can cause oncogenic effect on cells. Nonviral, NanoScript technology, comprising utility of transcription factors (TFs) functionalized gold nanoparticles, can target specific regulatory site in the genome effectively and direct differentiation of MSCs into another cell fate, depending on regime of TFs. For example, myogenic regulatory factor containing NanoScript-MRF differentiates the adipose tissue derived MSCs into muscle cells [92]. The multipotency characteristics represent MSCs as promising candidate for obtaining stable tissue constructs through coaxed 3D organoid culture; however heterogeneous distribution of MSCs slows down cell proliferation, rendering therapeutic applications of MSCs. Adopting two-step culture system for MSCs can yield homogeneous distribution of MSCs in biomaterial scaffolds. For example, fetal-MSCs coaxed in biomaterial when cultured first in rotating bioreactor followed with static culture lead to homogeneous distribution of MSCs in ECM components [7]. Occurrence of dental carries, periodontal disease, and tooth injury can impact individual's health, where bioengineering of teeth can be the alternative option. Coaxing of epithelial-MSCs with dental stem cells into synthetic polymer gives rise to mature teeth unit, which consisted of mature teeth and oral tissue, offering multiple regenerative therapeutics, reviewed elsewhere [52]. Like the tooth decay, both human and animals are prone to orthopedic injuries, affecting bones, joint, tendon, muscles, cartilage, and so forth. Although natural healing potential of bone is sufficient to heal the common injuries, severe trauma and tumor-recession can abrogate germinal potential of bone-forming stem cells. In vitro chondrogenic, osteogenic, and adipogenic potential of MSCs advocates therapeutic applications of MSCs in orthopedic injuries [53]. Seeding of MSCs, coaxed into biomaterial scaffolds, at defective bone tissue, regenerates defective bone tissues, within four wks of transplantation; by the end of 32 wks newly formed tissues integrate into old bone [54]. Osteoblasts, the bone-forming cells, have lesser actin cytoskeleton compared to adipocytes and MSCs. Treatment of MSCs with cytochalasin-D causes rapid transportation of G-actin, leading to osteogenic transformation of MSCs. Furthermore, injection of cytochalasin-D to mice tibia also promotes bone formation within a wk time frame [55]. The bone formation processes in mice, dog, and human are fundamentally similar, so outcomes of research on mice and dogs can be directional for regenerative application to human. Injection of MSCs to femur head of Legg-Calve-Perthes suffering dog heals the bone very fast and reduces the injury associated pain [55]. Degeneration of skeletal muscle and muscle cramps are very common to sledge dogs, animals, and individuals involved in adventurous athletics activities. Direct injection of adipose tissue derived MSCs to tear-site of semitendinosus muscle in dogs heals injuries much faster than traditional therapies [56]. Damage effect treatment for heart muscle regeneration is much more complex than regeneration of skeletal muscles, which needs high grade fine-tuned coordination of neurons with muscles. Coaxing of MSCs into alginate gel increases cell retention time that leads to releasing of tissue repairing factors in controlled manner. Transplantation of alginate encapsulated cells to mice heart reduces scar size and increases vascularisation, which leads to restoration of heart functions. Furthermore, transplanted MSCs face host inhospitable inflammatory immune responses and other mechanical forces at transplantation site, where encapsulation of cells keeps them away from all sorts of mechanical forces and enables sensing of host tissue microenvironment, and respond accordingly [57]. Ageing, disease, and medicine consumption can cause hair loss, known as alopecia. Although alopecia has no life threatening effects, emotional catchments can lead to psychological disturbance. The available treatments for alopecia include hair transplantation and use of drugs, where drugs are expensive to afford and generation of new hair follicle is challenging. Dermal papillary cells (DPCs), the specialized MSCs localized in hair follicle, are responsible for morphogenesis of hair follicle and hair cycling. The layer-by-layer coating of DPCs, called GAG coating, consists of coating of geletin as outer layer, middle layer of fibroblast growth factor 2 (FGF2) loaded alginate, and innermost layer of geletin. GAG coating creates tissue microenvironment for DPCs that can sustain immunological and mechanical obstacles, supporting generation of hair follicle. Transplantation of GAG-coated DPCs leads to abundant hair growth and maturation of hair follicle, where GAG coating serves as ECM, enhancing intrinsic therapeutic potential of DPCs [58]. During infection, the inflammatory cytokines secreted from host immune cells attract MSCs to the site of inflammation, which modulates inflammatory responses, representing MSCs as key candidate of regenerative medicine for infectious disease therapeutics. Coculture of macrophages (M*ϕ*) and adipose derived MSCs from* Leishmania major* (LM) susceptible and resistant mice demonstrates that AD-MSCs educate M*ϕ* against LM infection, differentially inducing M1 and M2 phenotype that represents AD-MSC as therapeutic agent for leishmanial therapy [93]. In summary, the multilineage differentiation potential of MSCs, as well as adoption of next-generation organoid culture system, avails MSCs as ideal regenerative medicine candidate.

## 5. UCSCs in Regenerative Medicine

Umbilical cord, generally thrown at the time of child birth, is the best known source for stem cells, procured in noninvasive manner, having lesser ethical constraints than ESCs. Umbilical cord is rich source of hematopoietic stem cells (HSCs) and MSCs, which possess enormous regeneration potential [94] (Figure 5; Table 1). The HSCs of cord blood are responsible for constant renewal of all types of blood cells and protective immune cells. The proliferation of HSCs is regulated by Musashi-2 protein mediated attenuation of Aryl hydrocarbon receptor (AHR) signalling in stem cells [95]. UCSCs can be cryopreserved at stem cells banks (Figure 5; Table 1), in operation by both private and public sector organization. Public stem cells banks operate on donation formats and perform rigorous screening for HLA typing and donated UCSCs remain available to anyone in need, whereas private stem cell banks operation is more personalized, availing cells according to donor consent. Stem cell banking is not so common, even in developed countries. Survey studies find that educated women are more eager to donate UCSCs, but willingness for donation decreases with subsequent deliveries, due to associated cost and safety concerns for preservation [96]. FDA has approved five HSCs for treatment of blood and other immunological complications [97]. The amniotic fluid, drawn during pregnancy for standard diagnostic purposes, is generally discarded without considering its vasculogenic potential. UCSCs are the best alternatives for those patients who lack donors with fully matched HLA typing for peripheral blood and PBMCs and bone marrow [98]. One major issue with UCSCs is number of cells in transplant, fewer cells in transplant require more time for engraftment to mature, and there are also risks of infection and mortality; in that case ex vivo propagation of UCSCs can meet the demand of desired outcomes. There are diverse protocols, available for ex vivo expansion of UCSCs, reviewed elsewhere [99]. Amniotic fluid stem cells (AFSCs), coaxed to fibrin (required for blood clotting, ECM interactions, wound healing, and angiogenesis) hydrogel and PEG supplemented with vascular endothelial growth factor (VEGF), give rise to vascularised tissue, when grafted to mice, suggesting that organoid cultures of UCSCs have promise for generation of biocompatible tissue patches, for treating infants born with congenital heart defects [59]. Retroviral integration of OCT4, KLF4, cMYC, and SOX2 transforms AFSCs into pluripotency stem cells known as AFiPSCs which can be directed to differentiate into extraembryonic trophoblast by BMP2 and BMP4 stimulation, which can be used for regeneration of placental tissues [60]. Wharton's jelly (WJ), the gelatinous substance inside umbilical cord, is rich in mucopolysaccharides, fibroblast, macrophages, and stem cells. The stem cells from UCB and WJ can be transdifferentiated into *β*-cells. Homogeneous nature of WJ-SCs enables better differentiation into *β*-cells; transplantation of these cells to streptozotocin induced diabetic mice efficiently brings glucose level to normal [7]. Easy access and expansion potential and plasticity to differentiate into multiple cell lineages represent WJ as an ideal candidate for regenerative medicine but cells viability changes with passages with maximum viable population at 5th-6th passages. So it is suggested to perform controlled expansion of WJ-MSCS for desired regenerative outcomes [9]. Study suggests that CD34^+^ expression leads to the best regenerative outcomes, with less chance of host-versus-graft rejection. In vitro expansion of UCSCs, in presence of StemRegenin-1 (SR-1), conditionally expands CD34^+^ cells [61]. In type I diabetic mellitus (T1DM), T-cell mediated autoimmune destruction of pancreatic *β*-cells occurs, which has been considered as tough to treat. Transplantation of WJ-SCs to recent onset-T1DM patients restores pancreatic function, suggesting that WJ-MSCs are effective in regeneration of pancreatic tissue anomalies [62]. WJ-MSCs also have therapeutic importance for treatment of T2DM. A non-placebo controlled phase I/II clinical trial demonstrates that intravenous and intrapancreatic endovascular injection of WJ-MSCs to T2DM patients controls fasting glucose and glycated haemoglobin through improvement of *β*-cells functions, evidenced by enhanced c-peptides and reduced inflammatory cytokines (IL-1*β* and IL-6) and T-cells counts [63]. Like diabetes, systematic lupus erythematosus (SLE) also can be treated with WJ-MSCs transplantation. During progression of SLE host immune system targets its own tissue leading to degeneration of renal, cardiovascular, neuronal, and musculoskeletal tissues. A non-placebo controlled follow-up study on 40 SLE patients demonstrates that intravenous infusion of WJ-MSC improves renal functions and decreases systematic lupus erythematosus disease activity index (SLEDAI) and British Isles Lupus Assessment Group (BILAG), and repeated infusion of WJ-MSCs protects the patient from relapse of the disease [64]. Sometimes, host inflammatory immune responses can be detrimental for HSCs transplantation and blood transfusion procedures. Infusion of WJ-MSC to patients, who had allogenic HSCs transplantation, reduces haemorrhage inflammation (HI) of bladder, suggesting that WJ-MSCs are potential stem cells adjuvant in HSCs transplantation and blood transfusion based therapies [100]. Apart from WJ, umbilical cord perivascular space and cord vein are also rich source for obtaining MSCs. The perivascular MSCs of umbilical cord are more primitive than WJ-MSCs and other MSCs from cord suggest that perivascular MSCs might be used as alternatives for WJ-MSCs for regenerative therapeutics outcome [101]. Based on origin, MSCs exhibit differential in vitro and in vivo properties and advocate functional characterization of MSCs, prior to regenerative applications. Emerging evidence suggests that UCSCs can heal brain injuries, caused by neurodegenerative diseases like Alzheimer's, Krabbe's disease, and so forth. Krabbe's disease, the infantile lysosomal storage disease, occurs due to deficiency of myelin synthesizing enzyme (MSE), affecting brain development and cognitive functions. Progression of neurodegeneration finally leads to death of babies aged two. Investigation shows that healing of peripheral nervous system (PNS) and central nervous system (CNS) tissues with Krabbe's disease can be achieved by allogenic UCSCs. UCSCs transplantation to asymptomatic infants with subsequent monitoring for 4–6 years reveals that UCSCs recover babies from MSE deficiency, improving myelination and cognitive functions, compared to those of symptomatic babies. The survival rate of transplanted UCSCs in asymptomatic and symptomatic infants was 100% and 43%, respectively, suggesting that early diagnosis and timely treatment are critical for UCSCs acceptance for desired therapeutic outcomes. UCSCs are more primitive than BMSCs, so perfect HLA typing is not critically required, representing UCSCs as an excellent source for treatment of all the diseases involving lysosomal defects, like Krabbe's disease, hurler syndrome, adrenoleukodystrophy (ALD), metachromatic leukodystrophy (MLD), Tay-Sachs disease (TSD), and Sandhoff disease [65]. Brain injuries often lead to cavities formation, which can be treated from neuronal parenchyma, generated ex vivo from UCSCs. Coaxing of UCSCs into human originated biodegradable matrix scaffold and in vitro expansion of cells in defined culture conditions lead to formation of neuronal organoids, within three wks' time frame. These organoids structurally resemble brain tissue and consisted of neuroblasts (GFAP^+^, Nestin^+^, and Ki67^+^) and immature stem cells (OCT4^+^ and SOX2^+^). The neuroblasts of these organoids further can be differentiated into mature neurons (MAP2^+^ and TUJ1^+^) [66]. Administration of high dose of drugs in divesting neuroblastoma therapeutics requires immediate restoration of hematopoiesis. Although BMSCs had been promising in restoration of hematopoiesis UCSCs are sparely used in clinical settings. A case study demonstrates that neuroblastoma patients who received autologous UCSCs survive without any associated side effects [12]. During radiation therapy of neoplasm, spinal cord myelitis can occur, although occurrence of myelitis is a rare event and usually such neurodegenerative complication of spinal cord occurs 6–24 years after exposure to radiations. Transplantation of allogenic UC-MSCs in laryngeal patients undergoing radiation therapy restores myelination [102]. For treatment of neurodegenerative disease like Alzheimer's disease (AD), amyotrophic lateral sclerosis (ALS), traumatic brain injuries (TBI), Parkinson's, SCI, stroke, and so forth, distribution of transplanted UCSCs is critical for therapeutic outcomes. In mice and rat, injection of UCSCs and subsequent MRI scanning show that transplanted UCSCs migrate to CNS and multiple peripheral organs [67]. For immunomodulation of tumor cells disease recovery, transplantation of allogenic DCs is required. The CD11c^+^DCs, derived from UCB, are morphologically and phenotypically similar to those of peripheral blood derived CTLs-DCs, suggesting that UCB-DCs can be used for personalized medicine of cancer patient, in need for DCs transplantation [103]. Coculture of UCSCs with radiation exposed human lung fibroblast stops their transdifferentiation, which suggests that factors secreted from UCSCs may restore niche identity of fibroblast, if they are transplanted to lung after radiation therapy [104]. Tearing of shoulder cuff tendon can cause severe pain and functional disability, whereas ultrasound guided transplantation of UCB-MSCs in rabbit regenerates subscapularis tendon in four wks' time frame, suggesting that UCB-MSCs are effective enough to treat tendons injuries when injected to focal points of tear-site [68]. Furthermore, transplantation of UCB-MSCs to chondral cartilage injuries site in pig knee along with HA hydrogel composite regenerates hyaline cartilage [69], suggesting that UCB-MSCs are effective regenerative medicine candidate for treating cartilage and ligament injuries. Physiologically circulatory systems of brain, placenta, and lungs are similar. Infusion of UCB-MSCs to preeclampsia (PE) induced hypertension mice reduces the endotoxic effect, suggesting that UC-MSCs are potential source for treatment of endotoxin induced hypertension during pregnancy, drug abuse, and other kinds of inflammatory shocks [105]. Transplantation of UCSCs to severe congenital neutropenia (SCN) patients restores neutrophils count from donor cells without any side effect, representing UCSCs as potential alternative for SCN therapy, when HLA matched bone marrow donors are not accessible [106]. In clinical settings, the success of myocardial infarction (MI) treatment depends on ageing, systemic inflammation in host, and processing of cells for infusion. Infusion of human hyaluronan hydrogel coaxed UCSCs in pigs induces angiogenesis, decreases scar area, improves cardiac function at preclinical level, and suggests that the same strategy might be effective for human [107]. In stem cells therapeutics, UCSCs transplantation can be either autologous or allogenic. Sometimes, the autologous UCSCs transplants cannot combat over tumor relapse, observed in Hodgkin's lymphoma (HL), which might require second dose transplantation of allogenic stem cells, but efficacy and tolerance of stem cells transplant need to be addressed, where tumor replace occurs. A case study demonstrates that second dose allogenic transplants of UCSCs effective for HL patients, who had heavy dose in prior transplant, increase the long term survival chances by 30% [10]. Patients undergoing long term peritoneal renal dialysis are prone to peritoneal fibrosis and can change peritoneal structure and failure of ultrafiltration processes. The intraperitoneal (IP) injection of WJ-MSCs prevents methylglyoxal induced programmed cell death and peritoneal wall thickening and fibrosis, suggesting that WJ-MSCs are effective in therapeutics of encapsulating peritoneal fibrosis [70]. In summary, UCB-HSCs, WJ-MSCs, perivascular MSCs, and UCB-MSCs have tissue regeneration potential.

## 6. BMSCs in Regenerative Medicine

Bone marrow found in soft spongy bones is responsible for formation of all peripheral blood and comprises hematopoietic stem cells (producing blood cells) and stromal cells (producing fat, cartilage, and bones) [108] (Figure 6; Table 1). Visually bone marrow has two types, red marrow (myeloid tissue; producing RBC, platelets, and most of WBC) and yellow marrow (producing fat cells and some WBC) [108]. Imbalance in marrow composition can culminate to the diseased condition. Since 1980, bone marrow transplantation is widely accepted for cancer therapeutics [109]. In order to avoid graft rejection, HLA typing of donors is a must, but completely matched donors are limited to family members, which hampers allogenic transplantation applications. Since matching of all HLA antigens is not critically required, in that case defining the critical antigens for haploidentical allogenic donor for patients, who cannot find fully matched donor, might relieve from donor constraints. Two-step administration of lymphoid and myeloid BMSCs from haploidentical donor to the patients of aplastic anaemia and haematological malignancies reconstructs host immune system and the outcomes are almost similar to fully matched transplants, which recommends that profiling of critically important HLA is sufficient for successful outcomes of BMSCs transplantation. Haploidentical HLA matching protocol is the major process for minorities and others who do not have access to matched donor [71]. Furthermore, antigen profiling is not the sole concern for BMSCs based therapeutics. For example, restriction of HIV1 (human immune deficiency virus) infection is not feasible through BMSCs transplantation because HIV1 infection is mediated through CD4^+^ receptors, chemokine CXC motif receptor 4 (CXCR4), and chemokine receptor 5 (CCR5) for infecting and propagating into T helper (Th), monocytes, macrophages, and dendritic cells (DCs). Genetic variation in CCR2 and CCR5 receptors is also a contributory factor; mediating protection against infection has been reviewed elsewhere [110]. Engineering of hematopoietic stem and progenitor cells (HSPCs) derived CD4^+^ cells to express HIV1 antagonistic RNA, specifically designed for targeting HIV1 genome, can restrict HIV1 infection, through immune elimination of latently infected CD4^+^ cells. A single dose infusion of genetically modified (GM), HIV1 resistant HSPCs can be the alternative of HIV1 retroviral therapy. In the present scenario stem cells source, patient selection, transplantation-conditioning regimen, and postinfusion follow-up studies are the major factors, which can limit application of HIV1 resistant GM-HSPCs (CD4^+^) cells application in AIDS therapy [72, 73]. Platelets, essential for blood clotting, are formed from megakaryocytes inside the bone marrow [74]. Due to infection, trauma, and cancer, there are chances of bone marrow failure. To an extent, spongy bone marrow microenvironment responsible for lineage commitment can be reconstructed ex vivo [75]. The ex vivo constructed 3D-scaffolds consisted of microtubule and silk sponge, flooded with chemically defined organ culture medium, which mimics bone marrow environment. The coculture of megakaryocytes and embryonic stem cells (ESCs) in this microenvironment leads to generation of functional platelets from megakaryocytes [75]. The ex vivo 3D-scaffolds of bone microenvironment can stride the path for generation of platelets in therapeutic quantities for regenerative medication of burns [75] and blood clotting associated defects. Accidents, traumatic injuries, and brain stroke can deplete neuronal stem cells (NSCs), responsible for generation of neurons, astrocytes, and oligodendrocytes. Brain does not repopulate NSCs and heal traumatic injuries itself and transplantation of BMSCs also can heal neurodegeneration alone. Lipoic acid (LA), a known pharmacological antioxidant compound used in treatment of diabetic and multiple sclerosis neuropathy when combined with BMSCs, induces neovascularisation at focal cerebral injuries, within 8 wks of transplantation. Vascularisation further attracts microglia and induces their colonization into scaffold, which leads to differentiation of BMSCs to become brain tissue, within 16 wks of transplantation. In this approach, healing of tissue directly depends on number of BMSCs in transplantation dose [76]. Dental caries and periodontal disease are common craniofacial disease, often requiring jaw bone reconstruction after removal of the teeth. Traditional therapy focuses on functional and structural restoration of oral tissue, bone, and teeth rather than biological restoration, but BMSCs based therapies promise for regeneration of craniofacial bone defects, enabling replacement of missing teeth in restored bones with dental implants. Bone marrow derived CD14^+^ and CD90^+^ stem and progenitor cells, termed as tissue repair cells (TRC), accelerate alveolar bone regeneration and reconstruction of jaw bone when transplanted in damaged craniofacial tissue, earlier to oral implants. Hence, TRC therapy reduces the need of secondary bone grafts, best suited for severe defects in oral bone, skin, and gum, resulting from trauma, disease, or birth defects [77]. Overall, HSCs have great value in regenerative medicine, where stem cells transplantation strategies explore importance of niche in tissue regeneration. Prior to transplantation of BMSCs, clearance of original niche from target tissue is necessary for generation of organoid and organs without host-versus-graft rejection events. Some genetic defects can lead to disorganization of niche, leading to developmental errors. Complementation with human blastocyst derived primary cells can restore niche function of pancreas in pigs and rats, which defines the concept for generation of clinical grade human pancreas in mice and pigs [111]. Similar to other organs, diaphragm also has its own niche. Congenital defects in diaphragm can affect diaphragm functions. In the present scenario functional restoration of congenital diaphragm defects by surgical repair has risk of reoccurrence of defects or incomplete restoration [8]. Decellularization of donor derived diaphragm offers a way for reconstruction of new and functionally compatible diaphragm through niche modulation. Tissue engineering technology based decellularization of diaphragm and simultaneous perfusion of bone marrow mesenchymal stem cells (BM-MSCs) facilitates regeneration of functional scaffolds of diaphragm tissues [8]. In vivo replacement of hemidiaphragm in rats with reseeded scaffolds possesses similar myography and spirometry as it has in vivo in donor rats. These scaffolds retaining natural architecture are devoid of immune cells, retaining intact extracellular matrix that supports adhesion, proliferation, and differentiation of seeded cells [8]. These findings suggest that cadaver obtained diaphragm, seeded with BM-MSCs, can be used for curing patients in need for restoration of diaphragm functions (Figure 6; Table 1). However, BMSCs are heterogeneous population, which might result in differential outcomes in clinical settings; however clonal expansion of BMSCs yields homogenous cells population for therapeutic application [8]. One study also finds that intracavernous delivery of single clone BMSCs can restore erectile function in diabetic mice [112] and the same strategy might be explored for adult human individuals. The infection of hepatitis C virus (HCV) can cause liver cirrhosis and degeneration of hepatic tissue. The intraparenchymal transplantation of bone marrow mononuclear cells (BMMNCs) into liver tissue decreases aspartate aminotransferase (AST), alanine transaminase (ALT), bilirubin, CD34, and *α*-SMA, suggesting that transplanted BMSCs restore hepatic functions through regeneration of hepatic tissues [113]. In order to meet the growing demand for stem cells transplantation therapy, donor encouragement is always required [8]. The stem cells donation procedure is very simple; with consent donor gets an injection of granulocyte-colony stimulating factor (G-CSF) that increases BMSCs population. Bone marrow collection is done from hip bone using syringe in 4-5 hrs, requiring local anaesthesia and within a wk time frame donor gets recovered donation associated weakness.

## 7. iPSCs in Regenerative Medicine

The field of iPSCs technology and research is new to all other stem cells research, emerging in 2006 when, for the first time, Takahashi and Yamanaka generated ESCs-like cells through genetic incorporation of four factors, Sox2, Oct3/4, Klf4, and c-Myc, into skin fibroblast [3]. Due to extensive nuclear reprogramming, generated iPSCs are indistinguishable from ESCs, for their transcriptome profiling, epigenetic markings, and functional competence [3], but use of retrovirus in transdifferentiation approach has questioned iPSCs technology. Technological advancement has enabled generation of iPSCs from various kinds of adult cells phasing through ESCs or direct transdifferentiation. This section of review outlines most recent advancement in iPSC technology and regenerative applications (Figure 7; Table 1). Using the new edge of iPSCs technology, terminally differentiated skin cells directly can be transformed into kidney organoids [114], which are functionally and structurally similar to those of kidney tissue in vivo. Up to certain extent kidneys heal themselves; however natural regeneration potential cannot meet healing for severe injuries. During kidneys healing process, a progenitor stem cell needs to become 20 types of cells, required for waste excretion, pH regulation, and restoration of water and electrolytic ions. The procedure for generation of kidney organoids ex vivo, containing functional nephrons, has been identified for human. These ex vivo kidney organoids are similar to fetal first-trimester kidneys for their structure and physiology. Such kidney organoids can serve as model for nephrotoxicity screening of drugs, disease modelling, and organ transplantation. However generation of fully functional kidneys is a far seen event with today's scientific technologies [114]. Loss of neurons in age-related macular degeneration (ARMD) is the common cause of blindness. At preclinical level, transplantation of iPSCs derived neuronal progenitor cells (NPCs) in rat limits progression of disease through generation of 5-6 layers of photoreceptor nuclei, restoring visual acuity [78]. The various approaches of iPSCs mediated retinal regeneration including ARMD have been reviewed elsewhere [79]. Placenta, the cordial connection between mother and developing fetus, gets degenerated in certain pathophysiological conditions. Nuclear programming of OCT4 knock-out (KO) and wild type (WT) mice fibroblast through transient expression of GATA3, EOMES, TFAP2C, and +/− cMYC generates transgene independent trophoblast stem-like cells (iTSCs), which are highly similar to blastocyst derived TSCs for DNA methylation, H3K7ac, nucleosome deposition of H2A.X, and other epigenetic markings. Chimeric differentiation of iTSCs specifically gives rise to haemorrhagic lineages and placental tissue, bypassing pluripotency phase, opening an avenue for generation of fully functional placenta for human [115]. Neurodegenerative disease like Alzheimer's and obstinate epilepsies can degenerate cerebrum, controlling excitatory and inhibitory signals of the brain. The inhibitory tones in cerebral cortex and hippocampus are accounted by *γ*-amino butyric acid secreting (GABAergic) interneurons (INs). Loss of these neurons often leads to progressive neurodegeneration. Genomic integration of Ascl1, Dlx5, Foxg1, and Lhx6 to mice and human fibroblast transforms these adult cells into GABAergic-INs (iGABA-INs). These cells have molecular signature of telencephalic INs, release GABA, and show inhibition to host granule neuronal activity [81]. Transplantation of these INs in developing embryo cures from genetic and acquired seizures, where transplanted cells disperse and mature into functional neuronal circuits as local INs [82]. Dorsomorphin and SB-431542 mediated inhibition of TGF-*β* and BMP signalling direct transformation of human iPSCs into cortical spheroids. These cortical spheroids consisted of both peripheral and cortical neurons, surrounded by astrocytes, displaying transcription profiling and electrophysiology similarity with developing fetal brain and mature neurons, respectively [83]. The underlying complex biology and lack of clear etiology and genetic reprogramming and difficulty in recapitulation of brain development have barred understanding of pathophysiology of autism spectrum disorder (ASD) and schizophrenia. 3D organoid cultures of ASD patient derived iPSC generate miniature brain organoid, resembling fetal brain few months after gestation. The idiopathic conditions of these organoids are similar with brain of ASD patients; both possess higher inhibitory GABAergic neurons with imbalanced neuronal connection. Furthermore these organoids express forkhead Box G1 (FOXG1) much higher than normal brain tissue, which explains that FOXG1 might be the leading cause of ASD [84]. Degeneration of other organs and tissues also has been reported, like degeneration of lungs which might occur due to tuberculosis infection, fibrosis, and cancer. The underlying etiology for lung degeneration can be explained through organoid culture. Coaxing of iPSC into inert biomaterial and defined culture leads to formation of lung organoids that consisted of epithelial and mesenchymal cells, which can survive in culture for months. These organoids are miniature lung, resemble tissues of large airways and alveoli, and can be used for lung developmental studies and screening of antituberculosis and anticancer drugs [87]. The conventional multistep reprogramming for iPSCs consumes months of time, while CRISPER-Cas9 system based episomal reprogramming system that combines two steps together enables generation of ESCs-like cells in less than two wks, reducing the chances of culture associated genetic abrasions and unwanted epigenetic [80]. This approach can yield single step ESCs-like cells in more personalized way from adults with retinal degradation and infants with severe immunodeficiency, involving correction for genetic mutation of OCT4 and DNMT3B [80]. The iPSCs expressing anti-CCR5-RNA, which can be differentiated into HIV1 resistant macrophages, have applications in AIDS therapeutics [88]. The diversified immunotherapeutic application of iPSCs has been reviewed elsewhere [89]. The *α*-1 antitrypsin deficiency (A1AD) encoded by serpin peptidase inhibitor clade A member 1 (SERPINA1) protein synthesized in liver protects lungs from neutrophils elastase, the enzyme causing disruption of lungs connective tissue. A1AD deficiency is common cause of both lung and liver disease like chronic obstructive pulmonary disease (COPD) and liver cirrhosis. Patient specific iPSCs from lung and liver cells might explain pathophysiology of A1AD deficiency. COPD patient derived iPSCs show sensitivity to toxic drugs which explains that actual patient might be sensitive in similar fashion. It is known that A1AD deficiency is caused by single base pair mutation and correction of this mutation fixes the A1AD deficiency in hepatic-iPSCs [85]. The high order brain functions, like emotions, anxiety, sleep, depression, appetite, breathing heartbeats, and so forth, are regulated by serotonin neurons. Generation of serotonin neurons occurs prior to birth, which are postmitotic in their nature. Any sort of developmental defect and degeneration of serotonin neurons might lead to neuronal disorders like bipolar disorder, depression, and schizophrenia-like psychiatric conditions. Manipulation of Wnt signalling in human iPSCs in defined culture conditions leads to an in vitro differentiation of iPSCs to serotonin-like neurons. These iPSCs-neurons primarily localize to rhombomere 2-3 segment of rostral raphe nucleus, exhibit electrophysiological properties similar to serotonin neurons, express hydroxylase 2, the developmental marker, and release serotonin in dose and time dependent manner. Transplantation of these neurons might cure from schizophrenia, bipolar disorder, and other neuropathological conditions [116]. The iPSCs technology mediated somatic cell reprogramming of ventricular monocytes results in generation of cells, similar in morphology and functionality with PCs. SA note transplantation of PCs to large animals improves rhythmic heart functions. Pacemaker needs very reliable and robust performance so understanding of transformation process and site of transplantation are the critical aspect for therapeutic validation of iPSCs derived PCs [28]. Diabetes is a major health concern in modern world, and generation of *β*-cells from adult tissue is challenging. Direct reprogramming of skin cells into pancreatic cells, bypassing pluripotency phase, can yield clinical grade *β*-cells. This reprogramming strategy involves transformation of skin cells into definitive endodermal progenitors (cDE) and foregut like progenitor cells (cPF) intermediates and subsequent in vitro expansion of these intermediates to become pancreatic *β*-cells (cPB). The first step is chemically complex and can be understood as nonepisomal reprogramming on day one with pluripotency factors (OCT4, SOX2, KLF4, and hair pin RNA against p53), then supplementation with GFs and chemical supplements on day seven (EGF, bFGF, CHIR, NECA, NaB, Par, and RG), and two weeks later (Activin-A, CHIR, NECA, NaB, and RG) yielding DE and cPF [86]. Transplantation of cPB yields into glucose stimulated secretion of insulin in diabetic mice defines that such cells can be explored for treatment of T1DM and T2DM in more personalized manner [86]. iPSCs represent underrated opportunities for drug industries and clinical research laboratories for development of therapeutics, but safety concerns might limit transplantation applications (Figure 7; Table 1) [117]. Transplantation of human iPSCs into mice gastrula leads to colonization and differentiation of cells into three germ layers, evidenced with clinical developmental fat measurements. The acceptance of human iPSCs by mice gastrula suggests that correct timing and appropriate reprogramming regime might delimit human mice species barrier. Using this fact of species barrier, generation of human organs in closely associated primates might be possible, which can be used for treatment of genetic factors governed disease at embryo level itself [118]. In summary, iPSCs are safe and effective for treatment of regenerative medicine.

## 8. Stem Cells in Wildlife Conservation

The unstable growth of human population threatens the existence of wildlife, through overexploitation of natural habitats and illegal killing of wild animals, leading many species to face the fate of being endangered and go for extinction. For wildlife conservation, the concept of creation of frozen zoo involves preservation of gene pool and germ plasm from threatened and endangered species (Figure 8). The frozen zoo tissue samples collection from dead or live animal can be DNA, sperms, eggs, embryos, gonads, skin, or any other tissue of the body [119]. Preserved tissue can be reprogrammed or transdifferentiated to become other types of tissues and cells, which opens an avenue for conservation of endangered species and resurrection of life (Figure 8). The gonadal tissue from young individuals harbouring immature tissue can be matured in vivo and ex vivo for generation of functional gametes. Transplantation of SSCs to testis of male from the same different species can give rise to spermatozoa of donor cells [120], which might be used for IVF based captive breeding of wild animals. The most dangerous fact in wildlife conservation is low genetic diversity, too few reproductively capable animals which cannot maintain adequate genetic diversity in wild or captivity. Using the edge of iPSC technology, pluripotent stem cells can be generated from skin cells. For endangered drill,* Mandrillus leucophaeus,* and nearly extinct white rhinoceros,* Ceratotherium simum cottoni*, iPSC has been generated in 2011 [121]. The endangered animal drill (*Mandrillus leucophaeus*) is genetically very close to human and often suffers from diabetes, while rhinos are genetically far removed from other primates. The progress in iPSCs, from the human point of view, might be transformed for animal research for recapturing reproductive potential and health in wild animals. However, stem cells based interventions in wild animals are much more complex than classical conservation planning and biomedical research has to face. Conversion of iPSC into egg or sperm can open the door for generation of IVF based embryo; those might be transplanted in womb of live counterparts for propagation of population. Recently, iPSCs have been generated for snow leopard (*Panthera uncia*), native to mountain ranges of central Asia, which belongs to cat family; this breakthrough has raised the possibilities for cryopreservation of genetic material for future cloning and other assisted reproductive technology (ART) applications, for the conservation of cat species and biodiversity. Generation of leopard iPSCs has been achieved through retroviral-system based genomic integration of OCT4, SOX2, KLF4, cMYC, and NANOG. These iPSCs from snow leopard also open an avenue for further transformation of iPSCs into gametes [122]. The in vivo maturation of grafted tissue depends both on age and on hormonal status of donor tissue. These facts are equally applicable to accepting host. Ectopic xenografts of cryopreserved testis tissue from Indian spotted deer (*Moschiola indica*) to nude mice yielded generation of spermatocytes [123], suggesting that one-day procurement of functional sperm from premature tissue might become a general technique in wildlife conservation. In summary, tissue biopsies from dead or live animals can be used for generation of iPSCs and functional gametes; those can be used in assisted reproductive technology (ART) for wildlife conservation.

## 9. Future Perspectives

The spectacular progress in the field of stem cells research represents great scope of stem cells regenerative therapeutics. It can be estimated that by 2020 or so we will be able to produce wide array of tissue, organoid, and organs from adult stem cells. Inductions of pluripotency phenotypes in terminally differentiated adult cells have better therapeutic future than ESCs, due to least ethical constraints with adult cells. In the coming future, there might be new pharmaceutical compounds; those can activate tissue specific stem cells, promote stem cells to migrate to the side of tissue injury, and promote their differentiation to tissue specific cells. Except few countries, the ongoing financial and ethical hindrance on ESCs application in regenerative medicine have more chance for funding agencies to distribute funding for the least risky projects on UCSCs, BMSCs, and TSPSCs from biopsies. The existing stem cells therapeutics advancements are more experimental and high in cost; due to that application on broad scale is not feasible in current scenario. In the near future, the advancements of medical science presume using stem cells to treat cancer, muscles damage, autoimmune disease, and spinal cord injuries among a number of impairments and diseases. It is expected that stem cells therapies will bring considerable benefits to the patients suffering from wide range of injuries and disease. There is high optimism for use of BMSCs, TSPSCs, and iPSCs for treatment of various diseases to overcome the contradictions associated with ESCs. For advancement of translational application of stem cells, there is a need of clinical trials, which needs funding rejoinder from both public and private organizations. The critical evaluation of regulatory guidelines at each phase of clinical trial is a must to comprehend the success and efficacy in time frame.

## Figures and Tables

**Table 1:** Application of stem cells in regenerative medicine: stem cells (ESCs, TSPSCs, MSCs, UCSCs, BMSCs, and iPSCs) have diverse applications in tissue regeneration and disease therapeutics.

SCs	Disease	Factors causing disease	Mode of stem cells application	Physiological and mechanistic aspects of stem cells therapeutics	Improvements in disease signatures & future use	References
ESCs	Spinal cord injuries	Infection, cancer, and accidents	ESCs transplantation to injury site	ESCs and secreted vasculogenic and neurogenic factor support tissue homing	Regeneration of spinal tissue and improved balance and sensation	[15]
	ARMD and glaucoma	Macular cones degeneration	ESCs-derived cones and RGCs transplantation to eye	COCO (activating TGF-*β*, BMP, and Wnt) & BRN3 (knock-in by CRISPER-Cas9) make ESCs become cones and RGCs form cells sheet & neuronal connection	Recovery from ARMD and macular defects & restoration of vision	[16, 17]
	Cardiovascular disease	Diabetes, drugs, genetic factor, and life style	ESCs-derived CMs & biomaterial coaxed ESCs	Cardiomyocytes express GCaMP3, secreting vasculogenic factors, and Tbox3 differentiates ESCs into SANPCs	Suppresses heart arrhythmias. CMs electrophysiologically integrate to heart as pacemaker	[18, 19, 28]
	Liver injuries	Toxins, drugs, genetic factors, and infection	Transplantation of ESCs-derived hepatocytes	ESCs-hepatocyte conversion is marked with expression of Cytp450, PXR, CYPA4&29, HNF4-*α*, and UGTA1; cells in transplant repopulate injured liver tissue	Regeneration of liver tissue can be used as model for screening of drugs	[20, 23, 24]
	Diabetes	Life style, heart defects, and genetics	Transplantation of ESCs-derived PPCs	Progenitors (CD24^+^, CD49^+^ & CD133^+^) differentiate into *β*-cells, secrete insulin, and express PDX1, GCK, and GLUT2	Improvement in glucose level and obesity can be used for treatment of T1DM and T2DM	[25, 26]
	Osteoarthritis	When cartilage tissue wears away	Transplantation of chondrocytes organoids	Chondrocytes (SOX9^+^ & collagen-II^+^) form cells aggregate & remain active for 12 wks at transplantation site	Regeneration of cartilage tissue can be used for treatment of injuries faced by athletes	[27]

TSPSCs	Diabetes	Life style and genetic factors	Transplantation of SCs derived PPCs organoid	PPCs need niche supported active FGF & Notch signalling to become *β*-cell	PPCs occupancy as *β*-cell can treat T1DM & T2DM	[25, 29, 30]
	Neurodental problems	Accidents, age, and genetic factors	Transplantation of DSPSCs as neurons	Neurons express nestin, GFAP, *β*III-tubulin, and L-type Ca^2+^ channels	Possible application in treatment of neurodental abnormalities	[31, 32]
	Acoustic problems	Age, noise, drugs, and infection	IESCs/IESCs-derived hair cells transplantation	*γ*-secretase shuts Notch by *β*-catenin & Atoh1 in lrg5^+^IESCs to be hair cells	Cochlear regeneration leads to restoration of acoustic functions	[34, 35]
	Intestinal degeneration	Genetic factors and food borne infections	IPCs derived crypt-villi organoid transplantation	M*ϕ*, myofibroblasts, and bacteria signals IPCs to be crypt-villi organoid tissue	Regeneration of goblet mucosa can treat intestinal defects	[36–38]
	Corneal diseases	Burns, genetics, and inflammation	LPSCs transplantation to corneal tissue	LPSCs in transplant marked by ABCB5 differentiate into mature cornea	Regeneration of corneal tissue might treat multiple eye disease	[39, 40]
	Muscular deformities	Infection, drugs, and autoimmunity	Transplantation of PEG fibrinogen coaxed MABs	PDGF from MABs attract vasculogenic and neurogenic cells to transplant site	Muscle fibril regeneration; skeletal muscle defects treatment	[41, 42]
	Eye disease & retinopathy	Toxins, burns, and genetic factors	AdSCs intravitreal transplantation	AdSCs from healthy donor produce higher vasoprotective factors	Restoration of vascularisation, diabetic retinopathy treatment	[44, 45]
	Cardiac dysfunctions	Age, genetic factors, and toxins	Systemic infusion of CA-AdSCs myocardium	CA-AdSCs to epithelium differentiation are superior to AdSCs	Regeneration of ischemic myocardium	[47, 48]

MSCs	Bladder deformities	Cystitis, cancer, and infection	Transplantation of BD-MSCs to bladder	BDMSCs (CD105^+^, CD73^+^, CD34^−^, and CD45^−^) with SIS heal bladder in 10 wks	Bladder regeneration from different origins MSCs	[50, 51]
	Dental problems	Infection, cancer age, and accidents	Transplants of EMSCs + DSCs biopolymer tissue	EMSC-DSCs and vasculogenic factors in biopolymer give rise to mature teeth units	Regeneration of oral tissue and application in periodontics	[31, 52]
	Bone degeneration	Injuries and tumor autoimmunity	Coaxed MSCs transplant & MSCs infusion	Actin modelling by cytochalasin-D transforms MSCs into osteoblasts	Regeneration of bones, reduction in injury pain	[53–55]
	Muscle degeneration	Genetic factors and work stress	Coaxed MSCs transplant and MSCs infusion	Alginate gel protects MSCs from immune attack and controls GFs release	Regeneration of heart scar and muscle tissue in controlled way	[56, 57]
	Alopecia	Age, disease, and medicine use	Transplantation of GAG-coated DPCs	GAG coating mimics ECM microenvironment, promoting DPCs regeneration	Regeneration of hair follicle for treatment of alopecia	[58]

UCSCs	Congenital heart defects	Developmental errors	Transplantation of fibrin coaxed AFSCs	Addition of VEFG to PEG coaxed AFSCs promotes organogenesis	Regeneration of tissue repair for treatment of heart defects	[59, 60]
	Diabetes	Life style and genetic factors	WJ-SCs, transplantation, and intravenous injection	WJ-factors & M*ϕ* differentiate WJ-SCs into *β*-cells, decreasing IL6 & IL1*β*	Improvement in function of *β*-cells leads to treatment of diabetes	[7, 9, 61–63]
	SLE	Autoimmunity	Intravenous infusion of WJ-SCs	WJ-SCs decrease SLEDAI & BILAG; reinfusion protects from disease relapse	Improvement in renal functions & stopping degeneration of tissues	[64]
	LSD & neurodegenerative diseases	Genetics, tumor, age, and life style	Allogenic UCSCs cells and biomaterial coaxed UCSCs organoids	Organoids consisted of neuroblasts (GFAP^+^, Nestin^+^, and Ki67^+^) & SCs (OCT4^+^, SOC2^+^); UCSCs recover from MSE deficiency and improve cognition	Treatment of Krabbe's disease, hurler syndrome, MLD, TSD, ALD, AD, ALS, SCI, SCI, TBI, Parkinson's, stroke, and so forth	[65–67]
	Cartilage and tendon injuries	Accident	Transplantation of UCB-SCs, UCB-SCs-HA gel	HA gel factors promote regeneration of hyaline cartilage & tendons in wks time	Recovery from tendons and cartilage injuries	[68, 69]
	Hodgkin's lymphoma	Genetic and environmental	Transplantation of UCSCs	Second dose infection of allogenic UCSCs improves patients life by 30%	Treatment of Hodgkin's lymphoma and other cancers	[10]
	Peritoneal fibrosis	Long term renal dialysis and fibrosis	WJ-SCs, transplantation by IP injection	WJ-SCs prevent programmed cells death and peritoneal wall thickness	Effective in treatment of encapsulating peritoneal fibrosis	[70]

BMSCs	Anaemia and blood cancer	Injury, genetics autoimmunity	Two-step infusion of lymphoid and myeloid	Haplo identical BMSCs can reconstruct immunity, which is major process for minority	Treatment of aplastic anaemia & haematological malignancies	[71]
	AIDS	HIV1 infection	Transplantation of HIV1 resistant CD4^+^ cells	Anti-HIV1 CD4^+^ cells express HIV1 anti-RNA, which restrict HIV infection	Treatment of AIDS as an alternative of antiretroviral	[72, 73]
	Blood clotting disorders	Lack of platelets	Transplantation of megakaryocyte organoids	GFs in silk sponge, microtubule 3D scaffolds mimic bone marrow	Therapeutics of burns and blood clotting diseases	[74, 75]
	Neurodegenerative diseases	Accidents, age, trauma, and stroke	Focal transplant of BMSCs with LA	LA^+^BMSCs induce neovascularisation that directs microglia for colonization	Treatment of neuronal damage disorders and cognitive restoration	[76]
	Orodental deformities	Trauma, disease, and birth defects	Bone marrow derived stem & progenitor (TRC)	CD14^+^ & CD90^+^ TRC accelerate alveolar jaw bone regeneration	Regeneration of defects in oral bone, skin, and gum	[77]
	Diaphragm abnormalities	Accidents & birth defects	Implantation of decellularized diaphragm	BMSCs niche perfused hemidiaphragm has similar myography & spirometry	Replacement therapy by donor derived niched diaphragm	[8]

iPSCs	Eye defects	Age, genetics, and birth defects	iPSCs derived NPCs transplantation	NPCs form 5-6 layers of photoreceptor nuclei, restoring visual acuity	Treatment of ARMD and other age-related eye defects	[78–80]
	Neurodegenerative disorders	Accidents, age, trauma, and stroke	iGABA-INs and cortical spheroid transplantation	(iGABA-INs) secrete GABA; FOX1G cause ASD, spheroid mimics to brain	ASD, Alzheimer's, seizer, and obstinate epilepsies treatment	[81–84]
	Liver & lung diseases	A1AD deficiency	Transplantation of A1AD mutation corrected iPSCs	A1AD is encoded by SERPINA1 in liver, and mutation leads to drugs sensitivity	Treatment of COPD causing lungs and liver degeneration	[85]
	Diabetes	Life style and genetic factors	iPSCs derived *β*-cells transplantation	Skin to *β*-cells reprogramming phase through cDE & cPF requires GPs	Treatment of T1DM and T2DM and insulin production	[86]
	Lung degeneration	Tuberculosis, cancer, and fibrosis	Biomaterial coaxed iPSCs transplantation	Miniature iPSCs lung resembles airways and alveoli, model drug testing	Regeneration of lung tissue	[87]
	SIDs and AIDS	Age, genetic factors, and infection	Transplantation of Oct4 and Nanog corrected iPSCs	CRISPER-Cas9 generate iPSCs in single step; iPSCs-M*ϕ* resists HIV1	Immunotherapy of SIDs, HIV1, and other immune diseases	[80, 88, 89]

## Abbreviations

ESCs: Embryonic stem cells
TSPSCs: Tissue specific progenitor stem cells
UCSCs: Umbilical cord stem cells
BMSCs: Bone marrow stem cells
iPSCs: Induced pluripotent stem cells
MSCs: Mesenchymal stem cells
WJ-MSCs: Wharton's jelly mesenchymal stem cells
HSCs: Hematopoietic stem cells
RGCs: Retinal ganglion cells
T1DM: Type I diabetes mellitus
T2DM: Type 2 diabetes mellitus
A1AD: *α*-1 antitrypsin deficiency
COPD: Chronic obstructive pulmonary disease
HLA: Human leukocyte antigen
MHC: Major histocompatibility complex
3D: Three-dimensional
SCI: Spinal cord injury
ARMD: Age-related macular degeneration
RPE: Retinal pigment epithelium
PXR: Pregnane X receptor
DPSCs: Dental pulp stem cells
GFAP: Glial fibrillary acidic protein
Atoh1: Activation of transcription factor atonal homologue 1
NEC: Necrotizing enterocolitis
DMD: Duchene muscular dystrophy
PDGF: Placental derived growth factor
PEG: Polyethylene glycol
SSCs: Spermatogonial stem cells
AdSCs: Adipose derived stem cells
HSCs: Hematopoietic stem cells
AFSCs: Amniotic fluid stem cells
VEGF: Vascular endothelial growth factor
UCB: Umbilical cord blood
SLEDAI: Systematic lupus erythematosus (SLE) disease activity index
HIV-1: Human immunodeficiency virus-1
GM-HSPCs: Genetically modified hematopoietic stem and progenitor cells
Th: T helper
LA: Lipoic acid
TRC: Tissue repair cells
BM-MSCs: Bone marrow mesenchymal stem cells
PBSCs: Peripheral blood stem cells
G-CSF: Granulocyte-colony stimulating factor
SERPINA1: Serpin peptidase inhibitor clade A member 1
ASD: Autism spectrum disorder
INs: Interneurons
GABAergic: *ϒ*-amino butyric acid secreting
NPCs: Neuronal progenitor cells
iTSCs: Independent trophoblast stem-like cells
hCS: Human cortical spheroids
CMs: Cardiomyocytes
ALD: Adrenoleukodystrophy
MLD: Metachromatic leukodystrophy
TSD: Tay-Sachs disease
ALS: Amyotrophic lateral sclerosis
TBI: Traumatic brain injuries
AD: Alzheimer's disease
NSCs: Neuronal stem cells
SID: Severe immune deficiency.
